# An iron metabolism and immune related gene signature for the prediction of clinical outcome and molecular characteristics of triple-negative breast cancer

**DOI:** 10.1186/s12885-022-09679-x

**Published:** 2022-06-07

**Authors:** Xiao-Fen Li, Wen-Fen Fu, Jie Zhang, Chuan-Gui Song

**Affiliations:** 1 Department of Breast Surgery, Fujian Medical University Union Hospital, Fuzhou, 350001 Fujian Province China; 2 Department of General Surgery, Fujian Medical University Union Hospital, Fuzhou, 350001 Fujian Province China; 3 Breast Cancer Institute, Fujian Medical University, Fuzhou, 350001 Fujian Province China

**Keywords:** Iron metabolism, Ferroptosis, Immune infiltration, Gene signature, Triple-negative breast cancer

## Abstract

**Background:**

An imbalance of intracellular iron metabolism can lead to the occurrence of ferroptosis. Ferroptosis can be a factor in the remodeling of the immune microenvironment and can affect the efficacy of cancer immunotherapy. How to combine ferroptosis-promoting modalities with immunotherapy to suppress triple-negative breast cancer (TNBC) has become an issue of great interest in cancer therapy. However, potential biomarkers related to iron metabolism and immune regulation in TNBC remain poorly understand.

**Methods:**

We constructed an optimal prognostic TNBC-IMRGs (iron metabolism and immune-related genes) signature using least absolute shrinkage and selection operator (LASSO) cox regression. Survival analysis and ROC curves were analyzed to identify the predictive value in a training cohort and external validation cohorts. The correlations of gene signature with ferroptosis regulators and immune infiltration are also discussed. Finally, we combined the gene signature with the clinical model to construct a combined model, which was further evaluated using a calibration curve and decision curve analysis (DCA).

**Results:**

Compared with the high-risk group, TNBC patients with low-risk scores had a remarkably better prognosis in both the training set and external validation sets. Both the IMRGs signature and combined model had a high predictive capacity, 1/3/5- year AUC: 0.866, 0.869, 0.754, and 1/3/5-yaer AUC: 0.942, 0.934, 0.846, respectively. The calibration curve and DCA also indicate a good predictive performance of the combined model. Gene set enrichment analysis (GSEA) suggests that the high-risk group is mainly enriched in metabolic processes, while the low-risk group is mostly clustered in immune related pathways. Multiple algorithms and single sample GSEA further show that the low-risk score is associated with a high tumor immune infiltration level. Differences in expression of ferroptosis regulators are also observed among different risk groups.

**Conclusions:**

The IMRGs signature based on a combination of iron metabolism and immune factors may contribute to evaluating prognosis, understanding molecular characteristics and selecting treatment options in TNBC.

**Supplementary information:**

The online version contains supplementary material available at 10.1186/s12885-022-09679-x.

## Background

Breast cancer, an extremely heterogeneous disease, is the most common cancer for women worldwide [1]. Triple-negative breast cancer (TNBC) is the most aggressive subtype of breast cancer with a high risk of distant relapse in the first 3 to 5 years following diagnosis [2]. More importantly, since there is no powerful targeted therapy currently available, the overall survival of TNBC patients is worse than patients with other subtypes of breast cancer [3, 4]. Therefore, identification of new sensitive biomarkers to determine prognosis or develop novel therapies for TNBC patients remains an urgent clinical need.

Iron is an essential trace element for the human body, and either an excess or shortage can influence many physiological and pathological processes including energy metabolism, mitochondrial function and DNA synthesis and repair [5]. It is reported that iron metabolism has dual effects in tumor growth and development. On the one hand, cancer cells are more dependent on iron intake for proliferation and more susceptible to iron deficiency than non-cancer cells, which is termed “iron addition” [6]. On the other hand, highly increased iron concentrations lead to cell death driven by excessive reactive oxygen species (ROS) and lipid peroxidation, known as ferroptosis [7]. Ferroptosis is a novel form of programmed cell death that is different from apoptosis, pyroptosis, necroptosis, and autophagy [8]. TNBC cells are more prone to ferroptosis due to their complex metabolic characteristics and cellular signaling pathways, which make it a promising choice to overcome the refractory disease [9].

An increasing amount of evidence indicates that immunotherapy can be an effective treatment strategy for TNBC patients [10]. Immune cells like macrophages and lymphocytes can play major roles in iron homeostasis through iron recycling of senescent erythrocytes [11] and non-transferrin bound iron uptake [12], respectively. In addition, a report has implicated a new mechanism by which CD8 + T cells repress tumor development through inducing ferroptosis [13]. Based on the crosstalk between iron metabolism and the tumor microenvironment (TME), it would be of significance to identify any potential applications of iron metabolism and immune related genes in targeted therapy and immunotherapy for TNBC patients.

Compared with a single biomarker, multigene signatures have been proven to produce a higher accuracy prognosis [14]. Previous studies have primarily focused on the prognostic value of the ferroptosis-related signature [15] and immune-related signature [16] in breast cancer. However, the identification of iron metabolism and immune-related signature in TNBC remains unclear.

The main purpose of this study was to identify the iron metabolism and immune-related genes (IMRGs) associated with the prognosis of TNBC through bioinformatics analysis, and then to build a prognosis model of TNBC patients according to LASSO regression analysis. The molecular characteristics of this model were identified by iron metabolism and tumor infiltrating lymphocytes. Finally, we combined the gene signature with the clinical model to construct a combined model, which had excellent predictive performance in terms of prognosis.

## Materials and methods

### Patient data sets

The gene expression profiles and clinical information of 119 normal samples and 123 TNBC samples were extracted from The Cancer Genome Atlas (TCGA) database (https://portal.gdc.cancer.gov/). The validation cohorts GSE2603 and GSE21653 (the available TNBC patient datasets with integral microarray data and survival data) were downloaded from the NCBI-GEOdatabase (https://www.ncbi.nlm.nih.gov/gds). There were no ethical conflicts because all data came from the public database.

### Identification of IMRGs

To meet the high demand of iron, cancer cells have remodeled iron metabolism pathways, including acquisition, storage, and efflux, affecting iron homeostasis [17]. Therefore, iron metabolism related genes were extracted from the gene sets “M15748: iron ion homeostasis”, “M11074: cellular iron ion homeostasis”, “M24450: cellular response to iron ion”, “M37743: abnormality of iron homeostasis”, “M18915: iron ion binding” and “M962: iron uptake and transport” from the Molecular Signatures Database (MSigDB). Ferroptosis related genes were obtained from the FerrDb database, “ferroptosis pathway (map04216)” from the KEGG PATHWAY Database [18] and “M39768: ferroptosis” from MSigDB. After integration of intersecting genes and elimination of unrelated genes, 499 iron metabolism and ferroptosis related genes were included in the follow-up studies. We also found 2483 immune related genes from the ImmPort database.

### Differential expressed analysis

Differential expressed genes (DEGs) of TNBC were identified between 119 normal samples and 123 TNBC samples with the cut-off values: |log_2_ fold change|> 1 and *P* < 0.05. Differently expressed genes were obtained between high-risk and low-risk groups with |log_2_ fold change|> 1 and *P* < 0.05 as the threshold. The results were calculated using the ‘limma’ package.

### Gene signature construction

The least absolute shrinkage and selection operator (LASSO) was used to construct the TNBC-IMRGs signature by TCGA transcriptome data. Risk score = -0.05076 × ExpEGR3-0.22386 × ExpCCND2-0.06754 × ExpSOCS3-0.06622 × ExpJunD-0.52219 × ExpSLC27A6.

### Functional enrichment analysis

KEGG/GO and GSEA were analyzed by the ‘clusterProfiler’ package [19]. The annotated gene sets of GSEA were selected, c2.cp.v7.2.symbols.gmt and c5.all.v7.2.symbols.gmt, from the MSigDB Collections (https://www.gsea-msigdb.org/gsea/msigdb/index.jsp), the number of permutations was set to 1000 times. The significance threshold was placed at FDR < 0.25, and *P*—value < 0.05.

### Immune infiltration analysis

The ESTIMATE algorithm was determined by the ‘estimate’ package (version 1.0.13) [20]. The association between risk score and immune cell infiltration levels were analyzed via TIMER [21], QUANTISEQ [22], MCPCOUNTER [23], XCELL [24], EPIC [25], and CIBERSPRT [26] algorithms from TIMER2.0 (https://cistrome.shinyapps.io/timer/). Single sample GSEA (ssGSEA) was performed with the ‘GSVA’ package (version 1.34.0) [27].

### Statistical analysis

All R packages were executed using R Studio software (version 4.0.5). The ‘ggplot2’ R package (version 3.3.3) was used to visualize the volcano plot and heatmap. A Pearson correlation was used to describe the correlation analysis. Univariate and multivariate Cox regression analysis were used to identify the prognostic factors. Kaplan–Meier survival curves were compared using the log-rank test. The ROC analysis was performed with the ‘pROC’ package. Significance was statistically considered at ***P < 0.001, **P < 0.01, *P < 0.05, and ns, *P* ≥ 0.05.

## Results

### Identification and validation of TNBC-IMRGs signature-based prognostic model

**Fig. 1:**
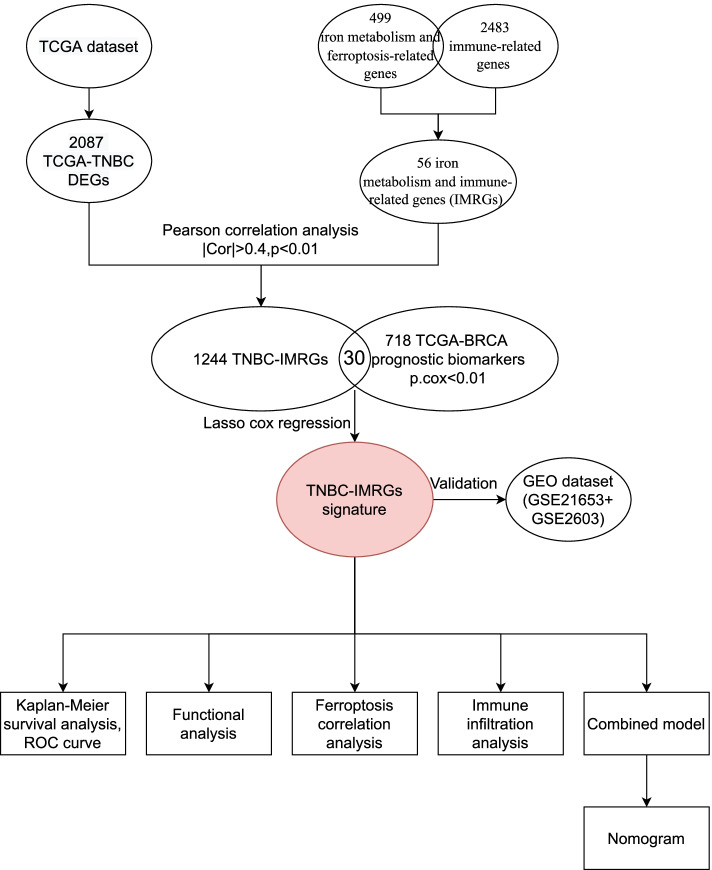
Study flow chart. Firstly, 2087 TNBC-DEGs and 56 IMRGs were identified. Next, a Pearson correlation analysis was performed between these two datasets and then we acquired 1244 TNBC-IMRGs. Subsequently, this dataset was integrated with the BRCA prognostic genes to obtain 30 candidate prognostic TNBC-IMRGs. Through LASSO analysis, a five-IMRGs signature was constructed. Survival analysis and ROC curve were performed to identify the prognostic value. Differences of molecular characteristics were evaluated between high- and low- risk groups. Finally, a combined model was constructed by combining the gene signature with the clinical variables

**Fig. 2:**
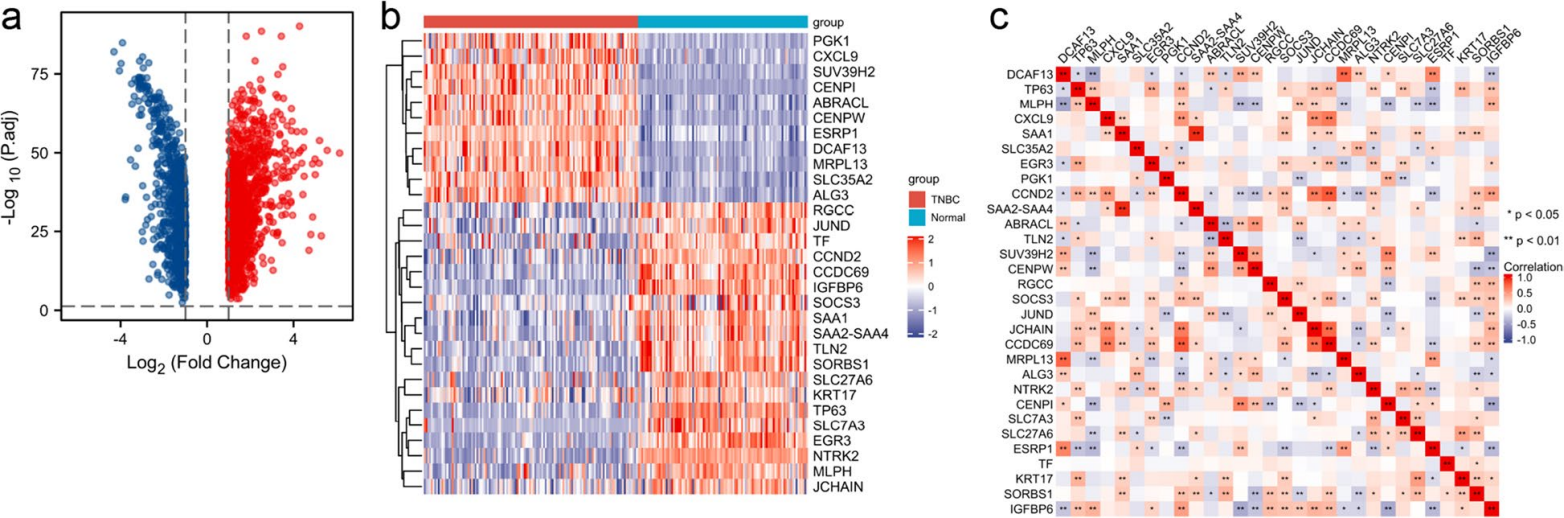
Thirty (30) candidate prognostic TNBC-IMRGs. **a** Volcano plot of TNBC-DEGs (|log_2_ fold change|> 1 and *P* < 0.05). Significantly upregulated and downregulated genes are depicted as red and blue dots, respectively. **b** Heatmap of expression profiles of 30 TNBC-IMRGs between TNBC and normal samples. **c** Correlation heatmap of 30 TNBC-IMRGs in expression levels

**Fig. 3:**
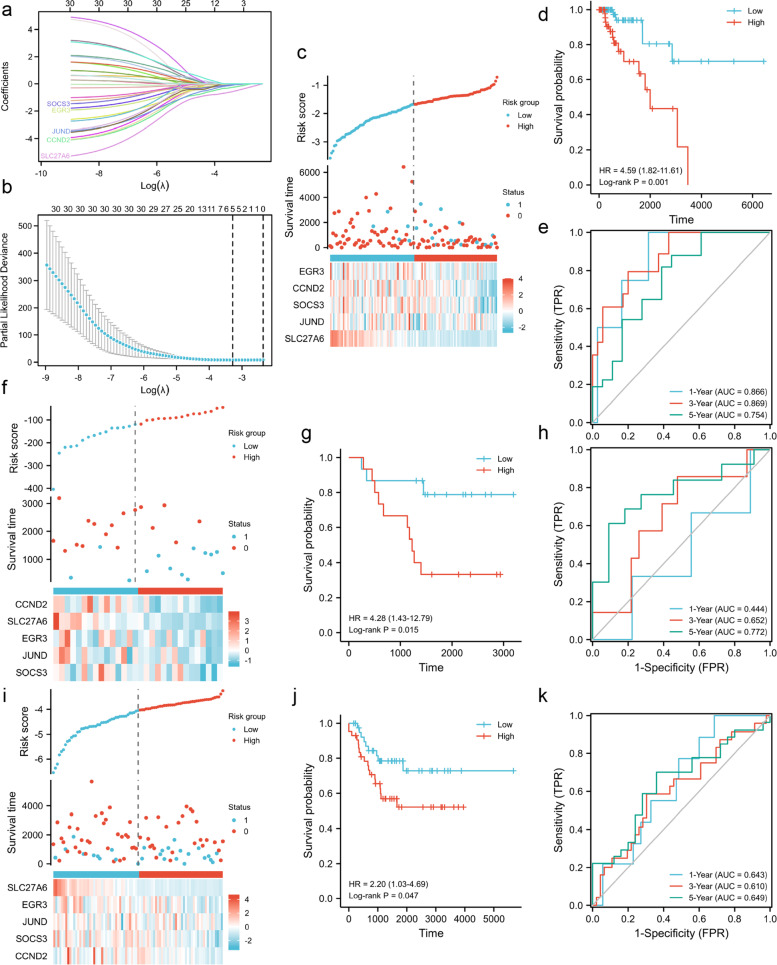
Identification and validation of TNBC-IMRGs signature. **a** The optimal lambda resulted in five nonzero coefficients. **b** Partial likelihood deviation curve was plotted. **c** Distribution of the risk score, survival status, and expression profiles of five genes in the TCGA training set. **d** Kaplan–Meier survival analysis of the high-risk group and low-risk group. **e** Time-dependent ROC curves at 1-, 3-, and 5-year of the TNBC-IMRGs signature for the training cohort. **f**, **i** Distribution of the risk score, survival status, and expression profiles of five genes in GSE2603 and GSE21653 validation set. **g-h**, **j-k** Kaplan–Meier survival analysis and ROC curves for the TNBC-IMRGs signature in the GSE2603 and GSE21653. Survival status: 1: dead, 0: alive. Low: low-risk group, High: high-risk group. Time: days. AUC: area under the curve

This study was conducted according to the flow chart presented in Fig. 1. The detailed clinicopathological features of TNBC patients from different cohorts are summarized in Table S1. A total of 2087 differently expressed genes (TNBC-DEGs) were identified between 119 normal samples and 123 TNBC samples from the TCGA dataset (Fig. 2a). We crossed over 499 iron metabolism and ferroptosis-related genes with 2483 immune-related genes to yielded 56 IMRGs. A Pearson correlation analysis was performed between TNBC-DEGs and IMRGs (Correlation Coefficient > 0.4 or < 0.4 and *P* < 0.01) and subsequently we acquired 1244 TNBC-IMRGs that had potential functions in both iron metabolism and tumor immunity. This dataset was integrated with the prognostic gene dataset from TCGA-BRCA (P.cox < 0.01) to obtain 30 candidate prognostic TNBC-IMRGs. The heatmap in Fig. 2b shows the expression profiles of these genes in TNBC and normal samples. Some TNBC-IMRGs show interrelationships in Fig. 2c. To further identify the best candidate genes, we performed LASSO cox regression to establish a TNBC-IMRGs signature-based prognostic model. When the model reached the minimum of lambda (λ), an optimal prognostic model with five non-zero coefficient genes (EGR3, CCND2, SOCS3, JunD, and SLC27A6) was constructed (Fig. 3a, 3b). A median risk score was calculated as follows: risk score = -0.05076 × ExpEGR3-0.22386 × ExpCCND2-0.06754 × ExpSOCS3-0.06622 × ExpJunD-0.52219 × ExpSLC27A6, and then we categorized patients into high-risk and low-risk groups. The distributions of the risk scores, survival time, survival status, and the expression patterns of five TNBC-IMRGs are displayed in Fig. 3c. As expected, all of these five protective genes were highly expressed in the low-risk group. Kaplan–Meier survival analysis showed that patients with low-risk scores exhibited a significantly better outcome than those with high-risk scores in the TCGA training set (*P* = 0.001, Fig. 3d). We used GSE2603 (Fig. 3f) and GSE21653 (Fig. 3i) as external validation sets to test the robustness of the model based on the TCGA-TNBC cohort. We consistently found that high-risk groups which were stratified using the same calculation formula as the training set were markedly related to worse prognosis in the validation sets (Fig. 3g, 3j). Next, through ROC curves, we evaluated the predictive accuracy of the prognostic model. The AUC of the TCGA cohort (Fig. 3e) at 1-, 3- and 5-years were 0.866, 0.869, and 0.754. The AUC at 1-, 3-, and 5-years of the GSE2603 cohort were 0.444, 0.652, and 0.772 (Fig. 3h) and for GSE21653 cohort were 0.643, 0.610, and 0.649 (Fig. 3k), respectively. These results indicate a very good prognostic value of this TNBC-IMRGs signature.

### Stratified prognostic analysis of the TNBC-IMRGs signature

**Fig. 4:**
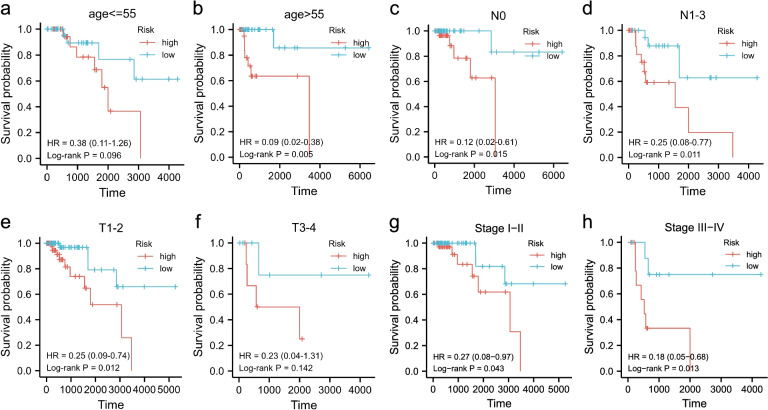
Stratified prognostic analysis of the TNBC-IMRGs signature. **a-h** The survival differences between high- and low- risk groups stratified by clinical variables: **a**, **b** age (≤ 55 and > 55), **c**, **d** node (N0 and N1-3), **e**, **f** tumor (T1-2 and T3-4), **g**, **h** stage (stage I-II and stage III-IV). Time: days

Stratified prognostic analysis suggests that the high-risk score is significantly associated with a worse OS in TNBC patients of an older age (> 55) (Figs. 4a-b) or smaller tumor size (T1-2) (Figs. 4e-f). Additionally, no matter the lymph node score (Figs. 4c-d) and stage status (Figs. 4g-h), patients with a low-risk score shown a better outcome than the high-risk group. This TNBC-IMRGs prognostic signature may act as an effective biomarker in assessing the prognosis of TNBC patients.

### Functional enrichment analysis and immune infiltration analysis

**Fig. 5:**
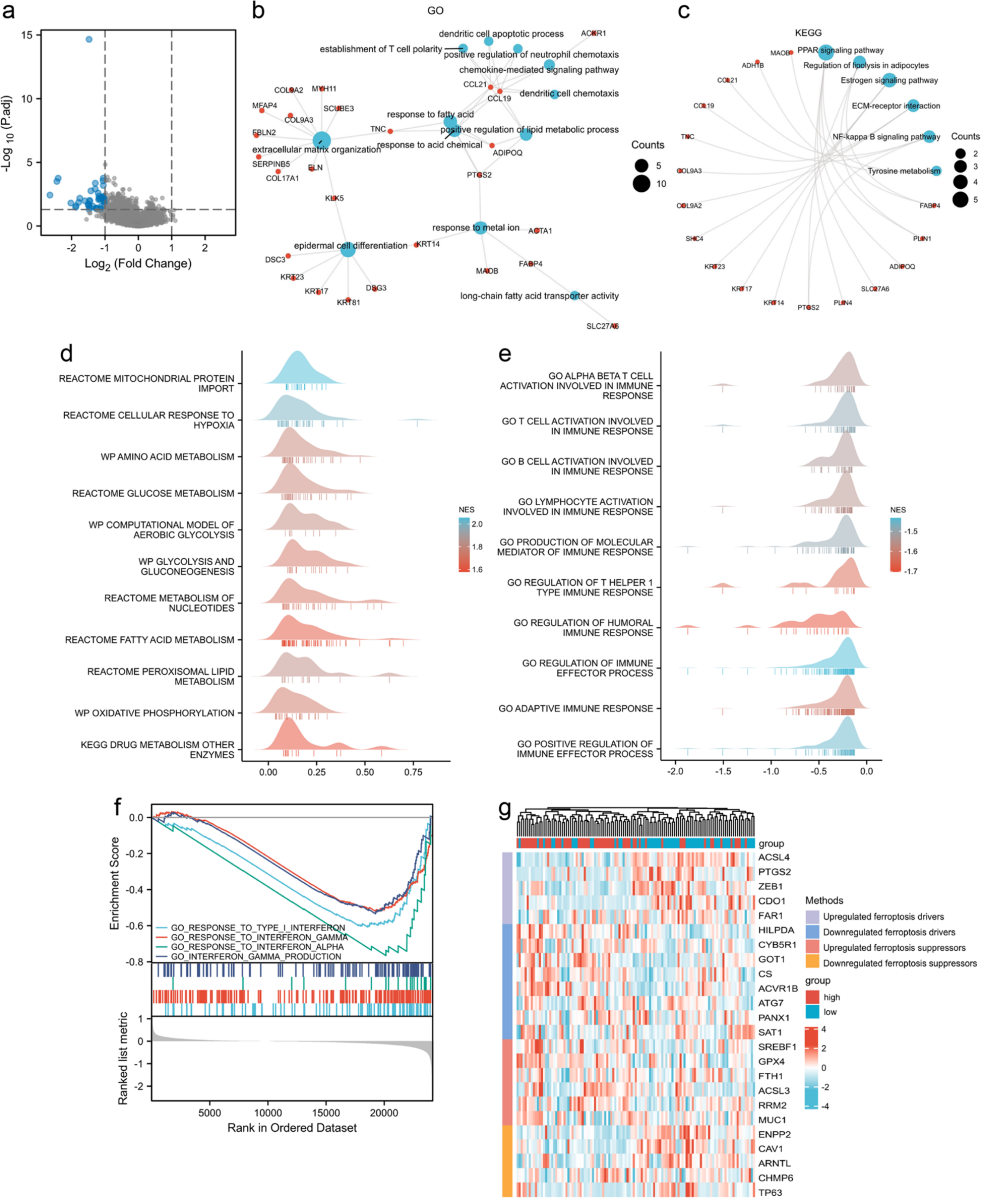
Functional enrichment analysis. **a** Volcano plot of DEGs between high- and low-risk groups (|log_2_ fold change|> 1 and *P* < 0.05). Significantly upregulated and downregulated genes are depicted as red and blue dots, respectively. **b-c** GO and KEGG enrichment analysis. **d-f** GSEA analysis for high- and low-risk groups. NES: normalize enrichment score. **g** Comparison of the differential expression of ferroptosis regulators in high- and low-risk groups

To explore the potential molecular characteristics in different risk groups, differently expressed genes between the high-risk group and low-risk group were screened out. The volcano map is depicted to visualize 44 DEGs (Fig. 5a). GO enrichment analysis revealed that the DEGs were significantly clustered in extracellular matrix organization, response to metal ions, positive regulation lipid metabolic process, establishment of T cell polarity, dendritic cell chemotaxis, and others (Fig. 5b). The KEGG pathway analysis demonstrated that most DEGs were clustered in physiological signaling pathways such as the PPAR signaling pathway, estrogen signaling pathway, NF-kappa B signaling pathway, etc. (Fig. 5c). Additionally, using GSEA analysis we found that multiple metabolism processes including cellular response to hypoxia, glucose metabolism, nucleotides and fatty acid metabolism, and oxidative phosphorylation were highly clustered in the high-risk group (Fig. 5d). While in the low-risk group, immune-related responses were enriched; for example, T cell, B cell, and lymphocyte activation, as well as the response to interferon (Fig. 5e, 5f).

Recent studies have already investigated a series of key regulatory genes that affect ferroptosis through abnormal accumulation of iron, free radicals, and ROS. Our results show that a total of four ferroptosis drivers (e.g., ACSL4 [28]) were upregulated in the low-risk group, while five anti-ferroptosis regulators (e.g., GPX4 [29], and FTH1 [30]) were overexpressed in the high-risk group (Fig. 5g).

**Fig. 6:**
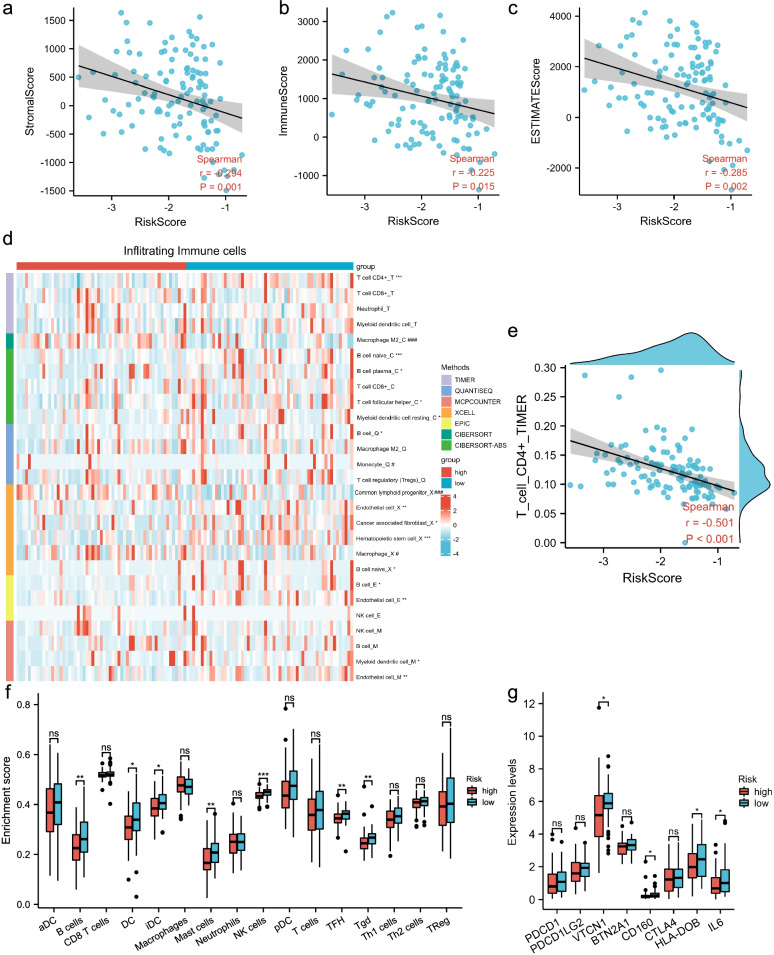
Immune infiltration analysis. **a-c** The correlation between risk score and immune related-scores with ESTIMATE algorithm. **d** Heatmap of infiltrating immune cells based on TIMER, QUANTISEQ, MCPCOUNTER, XCELL, EPIC, and CIBERSORT (* higher in low-risk group, *P < 0.05, **P < 0.01, ***P < 0.001; # higher in high-risk group, #*P* < 0.05, ###*P* < 0.001). **e** Scatter plot of the correlation between risk score and CD4 + T cell. **f** Enrichment scores of immune cells evaluated by ssGSEA. aDC: activated DC, iDC: immature DC, pDC: plasmacytoid DC, TFH: T follicular helper, Tgd: T gamma delta. **g** Differential expression analysis of immune checkpoint genes between high- and low-risk groups

Infiltrating stromal and immune cells, which make up the majority of normal cells in tumor tissue, not only interfere with tumor signaling but also regulate cancer biology. To further determine the relation of biological function with the TNBC-IMRGs signature in the immune response, we made use of various algorithms to investigate the immune microenvironment landscape. The ESTIMATE algorithm suggests that there might be a weak trend toward a negative correlation between risk score and immune-related score at the macro-levels (Figs. 6a-c). Therefore, we applied other algorithms, including TIMER, QUANTISEQ, MCPCOUNTER, EPIC, and CIBERSORT to further explore specific infiltrating immune cells (Fig. 6d). The results indicate that immunoreactive cells (like B cells, CD4 + T cells, and T follicular helper cells) were more abundant in the low-risk group, while immunosuppressive cells (e.g., M2 macrophage) were enriched in the high-risk group. There was a moderate negative correlation between CD4 + T cells and risk score (r = -0.501, P < 0.001) (Fig. 6e). Subsequently, differences were distinguished between the high- and low- risk groups in the quantities of 16 types of immune cells through ssGSEA (Fig. 6f). Similarly, the enrichment scores of immunoreactive cells (e.g., B cells, DC, iDC, Mast cells, NK cells, TFH, and Tgd) were higher in the low-risk group compared with the high-risk group (*P* < 0.05).

Immune checkpoint inhibitors are antitumor immunotherapies that are increasing used in clinical practice. Differences in immune checkpoint gene expression between high- and low-risk groups may confer differential susceptibility to immune checkpoint inhibitors. In our observations, we found that expression of some checkpoint molecules such as VTCN1, CD160, HLA-DOB, and IL6 in the low-risk group were all higher than that in the high-risk group (Fig. 6g). Taken together, these results suggest that the TNBC-IMRGs prognostic signature may be related to some extent to iron metabolism and immune cell infiltration.

### Construction and validation of the combined gene and clinical model

**Fig. 7:**
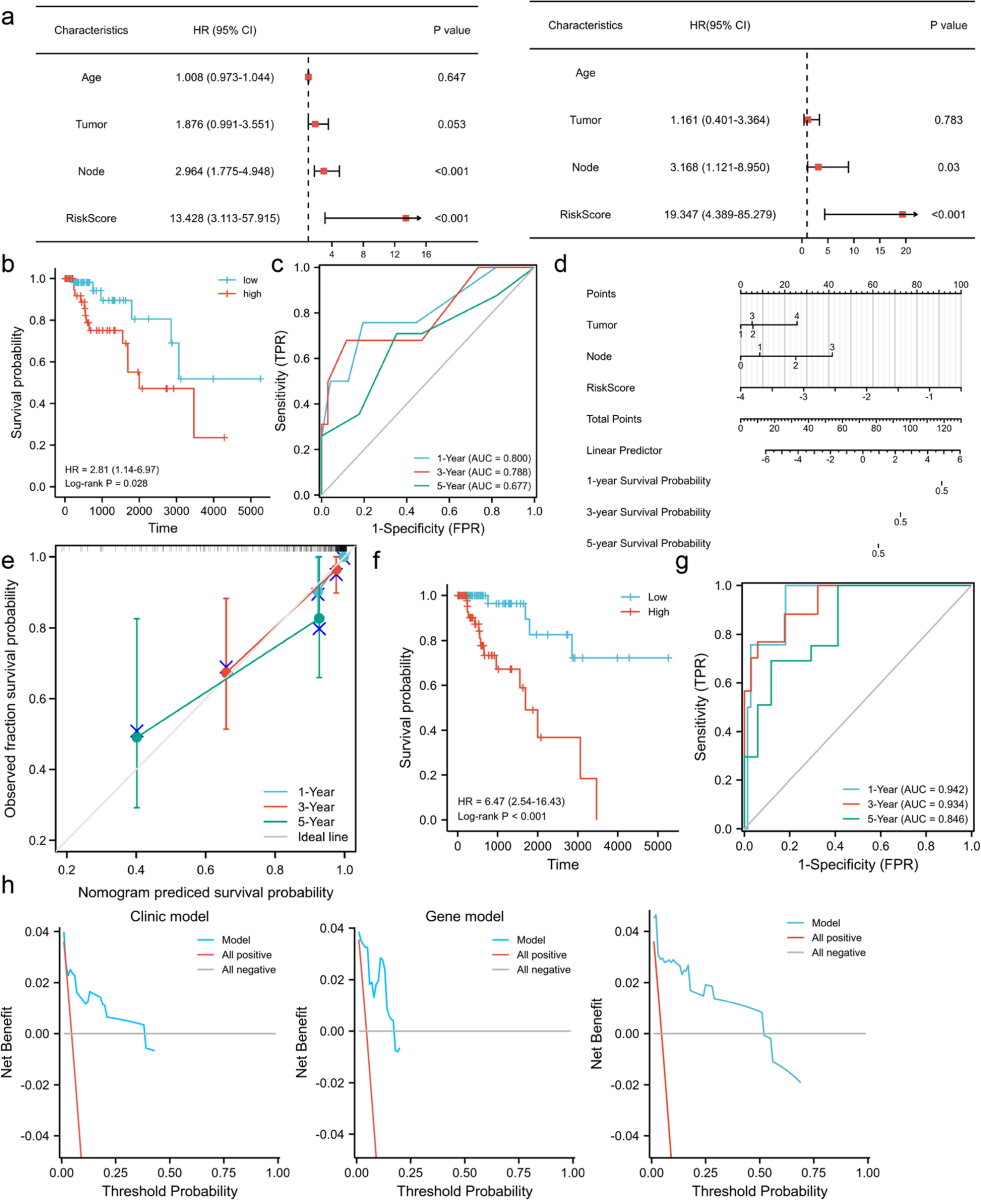
A predictive nomogram of the combined model was established. **a** Univariate and multivariate Cox regression analysis of the correlation between OS and various clinical variables including risk score. **b-c** Kaplan–Meier survival analysis **(b)** and ROC curves **(c)** in the clinical model. **d** The nomogram of the combined model for predicting the OS of patients with TNBC at 1-, 3-, and 5-year survival (Node stage and Tumor stage are categorical variables, Risk Score is a numeric variable). **e** Calibration plots of the nomogram at 1-, 3-, and 5-year survival. **f-g** Kaplan–Meier survival analysis **(f)** and ROC curves **(g)** in combined model. **h** Decision curve analysis of clinical model, gene signature and combined model. Low: low-risk group, High: high-risk group. Time: days. AUC: area under the curve

**Fig. 8:**
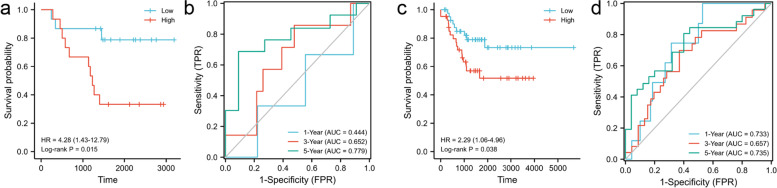
Validation of the combined model. **a-d** Kaplan–Meier survival analysis **(a, c)** and ROC curves **(b, d)** of the combined model in the validation sets GSE2603 and GSE21653

The forest plots are presented to illustrate the summary of the univariate and multivariate Cox regression analyses of some clinical features including risk score based on the TNBC-IMRGs signature (Fig. 7a). Node stage (*P* = 0.03) and risk score (*P* < 0.001) were determined to be robust independent prognostic predictors in the TCGA cohort. To further compare the superiority of gene-based signature, we constructed a clinical prognostic model that included Tumor stage and Node stage. The formula of the clinical risk score was as follows: clinical risk score = -1.18194 + 0.33509*Tumor stage + 1.01152*Node stage (Fig. 7b). The Fig. 7c depicts the AUC of the clinical model for 1-, 3-, and 5-year survival at 0.805, 0.791, and 0.657. It is obvious that the predictive power of the clinical model is not as good as the gene signature. We also established a combined model integrating the genomic risk score and clinical variables. The combined risk score was calculated as follows: combined risk score = 4.10626 + 0.32847*Tumor stage + 1.33369*Node stage + 2.94864*Risk Score (gene signature). The prognostic nomogram in the TCGA dataset is shown in Fig. 7d, and the C-index value for this combined model is 0.901. The combined model shows better prognostic performance than the gene signature and clinical model. There is a marked difference in prognosis between the high- and low-risk groups (Fig. 7f. *P* < 0.001). Likewise, the same trend is also observed in the validation sets; GSE2603 and GSE21653 (Fig. 8a, 8c). In addition, the AUC of the combined model for 1-, 3- and 5-year survival are 0.942, 0.934 and 0.846 (Fig. 7g). The calibration analysis of the 1-, 3- and 5- year outcome prediction is illustrated in Fig. 7e. The blue and red lines, not the green one, have a closer fit to the dotted gray line, revealing that the nomogram may do better predicting short-term prognosis than long-term prognosis. Additionally, decision curve analysis (DCA) indicates that the combined model has the best performance in prognosis prediction compared with the clinical model and gene signature (Fig. 7h). We also used two validation sets to evaluate this combined model. From Fig. 8, we can observe that the 1-, 3-, and 5-year AUC of the combined model in the GSE2603 set and GSE21653 set are higher than for the gene signature (0.444–0.779 vs. 0.444–0.772 and 0.657–0.735 vs. 0.610–0.649), which indicates the better prognostic performance of the combined model.

## Discussion

The imbalance of iron metabolism leads to excessive intracellular iron storage and the accumulation of lipid reactive oxygen species, which in turn induces ferroptosis. Intriguingly, some reports reveal that ferroptosis has been involved in the remodeling of the immune microenvironment and it can modify the efficacy of cancer immunotherapy. How to combine ferroptosis-promoting modalities with immunotherapy to produce a synergetic effect of tumor suppression has become a hot research topic. Recent evidence suggests that TNBC is particularly susceptible to ferroptosis, making it an attractive drug target. However, potential biomarkers related to both iron metabolism and immune regulation in TNBC have not been sufficiently examined.

There are already a variety of multigene signatures applied in breast cancer, for example Oncotype Dx® (21-genes) [31], Mammaprint (70-genes) [32], Breast Cancer Index (BCI) [33] and EndoPredict® (EP) [34]. However, the multigene prognostic model is poorly designed and validated in TNBC. As far as we know, our study is the first one to synthetically identify prognostic IMRGs in TNBC. We constructed a five-IMRGs signature and a combined prognostic model integrating the IMRGs signature with Tumor stage and Node stage to predict the likelihood of adverse events in TNBC patients. Compared with the high-risk group, TNBC patients with low-risk scores had remarkably better outcomes in both the training set and external validation sets. Both the IMRGs signature and the combined model had higher AUC values for 1-, 3- and 5-year survival than the clinical model.

TNBC cells are prone to ferroptosis, which is determined by intracellular metabolic processes that include lipid metabolism, iron metabolism, and amino acid metabolism [35, 36]. In our study, we found the high-risk group mostly enriched in various metabolic pathways (Fig. 5d). Apart from the common crucial regulatory genes of ferroptosis presented in Fig. 5g, five IMGRs (EGR3, SOCS3, JunD, SLC27A6, and CCND2) which were used to construct the gene signature were also shown to be partially involved in the process of ferroptosis. SOCS3, a member of the suppressor of cytokine signaling (SOCS) family, has been found to negatively regulate the upregulation of hepcidin which is a key regulator of iron metabolism [37]. More importantly, it has been shown to participate in tumor suppression and regulate ferroptosis by interacting with p53 [38]. Ferritin is a major cytosolic storage protein for iron that chelates excessive free iron, thereby reducing the occurrence of iron-mediated oxidative stress. Tsuji, Y reported that JunD was able to bind to the ferritin heavy chain and activate its transcription, which then protected cells from ROS in hepatocarcinoma cells [39]. Ni et al. found that cyclin D2(CCND2) downregulation promotes cardiomyocyte ferroptosis [40]. Whether EGR3 or SLC27A6 is involved in iron metabolism and ferroptosis has not been reported, our finding may provide clues for an underlying mechanism.

Since immune-related genes (EGR3 [41], CCND2 [42], SOCS3 [43], and JunD [44]) were also included in our IMRGs signature, we hypothesized that patients in different risk groups would have different proportion of infiltrating immune cells. Through various algorithms (Fig. 6), we found that the IMRGs risk scores tended to be negatively linked to immune cell infiltration levels, suggesting that increased IMRGs risk was associated with fewer infiltrating stromal and immune cells and higher tumor cells component [20]. In line with the tumor immunoediting hypothesis, some cancers may be eliminated by antitumor immune responses, whereas others develop due to evasion or partial control of immune surveillance [45]. Clearly, the high-risk group had a higher immunosuppression signature (e.g., M2 macrophage) and lower antitumor immune cell populations (e.g., B cells, NK cells, and T follicular helper cells) in the tumor microenvironment (Figs. 6d-f), which coincided with tumor progression and a worse prognosis for the high-risk group. Interestingly, most algorithms revealed that the B cell infiltration level was upregulated in the low-risk group (Fig. 6d). Lglesia et al. reported that the improved metastasis-free/progression-free survival was related to the B cell gene signature, mainly observed in basal-like and HER2-enriched breast cancer subtypes [46], which is in accordance with our results. We found that some immune checkpoint molecules expressed in immune cells (e.g., VTCN1 and CD160) were upregulated in the low-risk group, which may be driven by the increased infiltration of immune cells such as T cells and NK cells (Fig. 6g). However, due to the lower proportion of immunoreactive cells and lower expression of immune checkpoint genes, the high-risk group with a poorer prognosis may benefit less from immune checkpoint inhibitors than the low-risk group. A report has indicated that IFN-γ induces cell ferroptosis by activating the JAK1-2/STAT1/SLC7A11 signaling pathway. Meanwhile, treatment with IFN-γ reduces the level of GPX4, making cells more sensitive to ferroptosis [47]. In our data, we also found that IFN-γ related responses were enriched in the low-risk group (Fig. 5f). Maybe combination therapies with IFN-γ will be a basis for treatment of TNBC patients.

However, our study is not without limitations. Firstly, our study was mainly based on retrospective data of TCGA and GEO datasets, and prospective studies of the gene signature and combined model in multi-center cohorts will be necessary. Secondly, further biological experiments in vitro and vivo should be performed to reveal the potential mechanisms of IMRGs in TNBC.

## Conclusions

In conclusion, our study established a predictive signature associated with iron metabolism and tumor immunity that can accurately distinguish TNBC patients with different clinical outcomes. Moreover, this study provides evidence to support the combination of ferroptosis-induced cell death and antitumor immune in future clinical therapy in TNBC patients.

## Supplementary information

**Additional file 1:** **Table S1.** Clinical features of triple negative breast cancer in TCGA set, GSE2603 and GSE21653 set.

## Data Availability

The data supporting this study's findings are openly available in the TCGA dataset and GEO database.

## Abbreviations

TNBC: Triple-negative breast cancer
IMRGs: Iron metabolism and immune-related genes
ROS: Reactive oxygen species
TCGA: The Cancer Genome Atlas
GEO: Gene expression omnibus
DEGs: Differential expressed genes
LASSO: Least absolute shrinkage and selection operator
EGR3: Early growth response 3
SOCS3: Suppressor of cytokine signaling 3
JunD: JunD proto-oncogene
SLC27A6: Solute carrier family 27 member 6
CCND2: Cyclin D2
OS: Overall survival
ROC: Receiver operating characteristic
AUC: Area under the curve
DCA: Decision curve analysis
KEGG: Kyoto Encyclopedia of Genes and Genomes
GO: Gene Ontology
GSEA: Gene set enrichment analysis
ssGSEA: Single sample GSEA
TME: Tumor microenvironment
ACSL4: Acyl-CoA synthetase long chain family member 4
ACVR1B: Activin A receptor type 1B
FTH1: Ferritin heavy chain 1
GPX4: Glutathione peroxidase 4
IFN-γ: Interferon − gamma
TIMER 2.0: Tumor immune estimation resource, version 2
