# Supplementary material for: An iron metabolism and immune related gene signature for the prediction of clinical outcome and molecular characteristics of triple-negative breast cancer
Source: BMC Cancer. 2022 Jun 7;22:619. doi: 10.1186/s12885-022-09679-x (PMC9172128; doi:10.1186/s12885-022-09679-x)
Supplement: Supplementary file 1 — Additional file 1: Table S1. Clinical features of triple negative breast cancer in TCGA set, GSE2603 and GSE21653 set. [file 12885_2022_9679_MOESM1_ESM.docx]

Table 1. Clinical features of triple negative breast cancer in TCGA set, GSE2603 and GSE21653 set.

| Characteristics | TCGA-TNBC cohort (Training set) | GSE2603-TNBC  cohort (Validation set) | GSE21653-TNBC cohort (Validation set) |
| --- | --- | --- | --- |
| All of patients | 123 | 33 | 87 |
| **Age, years** |  |  |  |
| ≤55 | 67 (54.5%) | 16 (48.5%) | 42 (48.3%) |
| ＞55 | 56 (45.5%) | 17 (51.5%) | 45 (51.7%) |
| **T stage** |  |  |  |
| T1 | 26 (21.1%) | 6 (18.2%) | 16 (18.4%) |
| T2 | 82 (66.7%) | 19 (57.6%) | 46 (52.9%) |
| T3-4 | 14 (11.4%) | 8 (24.2%) | 23 (26.4%) |
| Unknown | 1 (0.8%) | 0 | 2 (2.3%) |
| **N stage** |  |  |  |
| N0 | 78 (63.4%) | 0 | 51 (58.6%) |
| N1-3 | 45 (36.6%) | 33 (100%) | 34 (39.1%) |
| Unknown | 0 | 0 | 2 (2.3%) |
| **M stage** |  |  |  |
| M0 | 122 (99.2%) | 17 (51.5%) | / |
| M1 | 1 (0.8%) | 13 (39.4%) | / |
| Unknown | 0 | 3 (9.1%) | / |
| **Stage** |  |  |  |
| I | 19 (15.4%) | / | / |
| II | 77 (62.6%) | / | / |
| III-IV | 23 (18.7%) | / | / |
| Unknown | 4 (3.3%) | / | / |
| **Menopausal state** |  |  |  |
| Pre | 34 (27.6%) | / | / |
| Peri | 5 (4.1%) | / | / |
| Post | 69 (56.1%) | / | / |
| Unknow | 15 (12.2%) | / | / |
